# Tumor Cells Develop Defined Cellular Phenotypes After 3D-Bioprinting in Different Bioinks

**DOI:** 10.3390/cells8101295

**Published:** 2019-10-22

**Authors:** Sonja K. Schmidt, Rafael Schmid, Andreas Arkudas, Annika Kengelbach-Weigand, Anja K. Bosserhoff

**Affiliations:** 1 Institute of Biochemistry, Emil-Fischer-Center, Friedrich-Alexander University of Erlangen-Nürnberg, 91054 Erlangen, Germany; Sonja.s.schmidt@fau.de; 2 Laboratory for Tissue-Engineering and Regenerative Medicine, Department of Plastic and Hand Surgery, University Hospital Erlangen—Friedrich Alexander University of Erlangen-Nürnberg FAU, 91054 Erlangen, Germany; Rafael.Schmid@uk-erlangen.de (R.S.); Andreas.arkudas@uk-erlangen.de (A.A.); Annika.kengelbach-weigand@uk-erlangen.de (A.K.-W.); 3 Comprehensive Cancer Center (CCC) Erlangen-EMN, 91054 Erlangen, Germany

**Keywords:** melanoma, 3D bioprinting, 3D cell culture, bioinks, biofabrication, tumor microenvironment, extracellular matrix

## Abstract

Malignant melanoma is often used as a model tumor for the establishment of novel therapies. It is known that two-dimensional (2D) culture methods are not sufficient to elucidate the various processes during cancer development and progression. Therefore, it is of major interest to establish defined biofabricated three-dimensional (3D) models, which help to decipher complex cellular interactions. To get an impression of their printability and subsequent behavior, we printed fluorescently labeled melanoma cell lines with Matrigel and two different types of commercially available bioinks, without or with modification (RGD (Arginine-Glycine-Aspartate)-sequence/laminin-mixture) for increased cell-matrix communication. In general, we demonstrated the printability of melanoma cells in all tested biomaterials and survival of the printed cells throughout 14 days of cultivation. Melanoma cell lines revealed specific differential behavior in the respective inks. Whereas in Matrigel, the cells were able to spread, proliferate and form dense networks throughout the construct, the cells showed no proliferation at all in alginate-based bioink. In gelatin methacrylate-based bioink, the cells proliferated in clusters. Surprisingly, the modifications of the bioinks with RGD or the laminin blend did not affect the analyzed cellular behavior. Our results underline the importance of precisely adapting extracellular matrices to individual requirements of specific 3D bioprinting applications.

## 1. Introduction

Regardless of constantly evolving possibilities for cancer treatments, there is still a huge demand for therapies offering a better outcome. In addition, methods for predicting the efficiency of novel approaches in patients are lacking. A commonly used model tumor for the development of new therapies is melanoma, a highly malignant tumor type derived from melanocytes and characterized by extremely early and fast metastasis from the primary tumor and subsequent fast progression. Many aspects of tumor formation and progression remain cryptic until today, hampering the development of novel approaches. This is not only due to the high complexity of the molecular processes within the biological systems but also owed to often simplified disease models, like two-dimensional (2D) cell culture, which are commonly used in basic research. For example, the existing 2D systems have helped to elucidate the six hallmarks of cancer [1] and support the process in developing cancer therapies. However, they are barely able to reflect more complex pathophysiological conditions, which is needed for future progress [2,3,4,5].

In their natural niche, cancer cells are subjected to a broad variety of cues coming from the extracellular matrix (ECM), stromal cells, vasculature and soluble factors [6]. With this in mind, researchers have started to establish additional three-dimensional (3D) cell culture systems, offering matrices to the cells, which are not only more physiological than stiff 2D culture dishes but can also be adjusted for individual approaches as desired [7,8]. Different methods are applied to design tumor models considering the 3D architecture. For example, tumor spheroids, which are widely used for drug testing, are tumor cell aggregates grown in medium or by using microfluidic devices without additional scaffolds [9]. Spheroids can be composed of a single tumor cell culture or cocultures of tumor cells with stromal cells to better mimic the complex interaction in the tumor mass. However, a major drawback of spheroids is the size. When it comes to the generation of large models, the lack of a scaffold becomes crucial for the agglomerated cells, and the spheroids tend to collapse due to undersupply of cells in the spheroid core [10]. To overcome the limitations in model size and also to design a near to in vivo microenvironment, tumor cells or even whole spheroids can be seeded onto scaffolds or can be embedded in hydrogels, which resemble the tumor microenvironment [10]. Although the design of appropriate matrix materials is not trivial, these methods enable the construction of model systems, taking different cell and material types into account [11]. The emerging field of biofabrication goes one step further, by using additive manufacturing, also known as 3D bioprinting, for the deposition of cells or whole spheroids within a desired matrix, thereby enabling uniform cell distribution and precise arrangement [12,13,14]. Therefore, the method of bioprinting gains more and more attention from researchers aiming to design highly defined 3D structures for basic research, tissue engineering and drug testing [15,16,17].

Matrices used for 3D bioprinting are required to comprise certain properties like cell compatibility, printability and mechanical stability [18]. For bioprinting and the reconstruction of ECM, the used materials are mostly hydrogels, which are mixed with the cells in a liquid or viscous precursor state, and get solidified through crosslinking after the printing process [19,20]. Common inks can be based on natural polymers like alginate or hyaluronic acid, synthetic polymers like polyethylene glycol (PEG) or even mixtures of both types [21].

Intriguingly, the basic composition, and with that, the physical and chemical properties of the matrices, can be adjusted as desired, and additionally, modifications with biologically active molecules like Arg-Gly-Asp (RGD)-peptides, laminins, fibronectins, etc., can be introduced in order to offer a certain range of cues for the embedded cells. There are several approaches using cell-type-specific modifications in order to mimic their physiological niche. For example, Wenz et al. used polymer solutions based on methacrylated gelatin and methacrylated hyaluronic acid modified with hydroxyapatite particles to mimic bone matrix and to trigger osteogenic differentiation of adipose-derived stem cells [22]. Wohlrab et al. used RGD-modified recombinant spider silk protein as a bioink and found the attachment and proliferation of BALB/3T3 mouse fibroblasts to be significantly improved in comparison to non-modified inks [23].

Here, we aimed to evaluate the printability of two different metastasis-derived melanoma cell lines with different commercially available matrices, which are supposed to be broadly usable for 3D bioprinting applications. For the first time, we deliver a proof of concept for the 3D bioprinting of melanoma cells in different materials, including Matrigel, noting the possibilities and limitations of working with application-independent bioinks. Our experiments revealed that the proliferation and morphology of cells in 3D-printed constructs depend on cell line and material composition, whereas in our case, ink modifications with an RGD-sequence or laminin mixture were not leading to cellular differences.

## 2. Materials and Methods

### 2.1. Cell Lines

The human melanoma cell line Mel Im [24] was stably transfected with GFP, as described by Hamm and colleagues [25], previously. The resulting cell line Mel Im GFP was cultured in Dulbecco’s Modified Eagle’s Medium (DMEM) low glucose, supplemented with 10% fetal calf serum (FCS), penicillin (400 U/mL) and streptomycin (50 µg/mL) (all from Sigma-Aldrich, St. Louis, MO, USA). Mel Im GFP were treated with Geneticin (200 µg/mL) (Gibco Life Technologies, Carlsbad, CA, USA) weekly to maintain GFP-expression. The human melanoma cell line MV3dc, stably expressing DsRed2 and histone-2B (H2B) eGFP, were a gift from P. Friedl’s group [26]. They were processed as described by Yamamoto et al. [27] and Alexander et al. [28] and cultured in DMEM high glucose, supplemented with 10% FCS, penicillin (400 U/mL) and streptomycin (50 µg/mL). MV3dc were treated with Geneticin (200 µg/mL) and Hygromycin (100 µg/mL) (Sigma Aldrich) weekly. Both cell lines were incubated at 37 °C in a humidified atmosphere containing 8% CO_2_.

### 2.2. Transient Transfection

We transiently transfected MV3 cells with a reporter for YAP/TAZ-TEAD complex activity. The reporter plasmid pGL4.23 MCAT-EGFP was a gift from Dr. Brabletz, Exp. Med.I, FAU Erlangen-Nürnberg [29]. For the transfection, 8 × 10^4^ cells were seeded into two wells of a six-well plate and transfected with 1 µg of reporter construct each, using Lipofectamine LTX (Invitrogen, Carlsbad, CA, USA), as described previously [30]. Four hours after transfection, cells were harvested and seeded into the biomaterials, as described below.

### 2.3. Three-Dimensional Bioprinting

For 3D bioprinting, the pneumatic extrusion-based bioprinter Cellink+ (Cellink, Goteborg, Sweden) was used.

Prior to printing, cell suspensions in the respective culture medium were mixed 1:11 with the respective hydrogel, *Cellink Bioink*, *Cellink RGD*, *GelXA* or *GelXA Laminink+* (all from Cellink) to a final concentration of 10^5^ cells/mL and filled into cartridges (Cellink). Grid patterns of 1 cm^2^, three layers high, were printed onto cover slips according to manufacturer protocols and crosslinked with Crosslinking Agent (Cellink), containing 50 mM CaCl_2_, for five minutes. Hardened constructs were washed with cell culture medium once and were transferred into six-well plates (Corning, New York City, NY, USA).

To print Matrigel, cells were mixed 1:11 with ice-cold Corning^®^ Matrigel^®^ Basement Membrane Matrix (Corning) to a final concentration of 10^5^ cells/mL and transferred into a cartridge. The cartridge was incubated at room temperature for 30 min to allow pre-gelling of the material. Constructs were printed on glass slides, which were transferred into six-well plates quickly, and were incubated at 37 °C for 30 min to thermally crosslink the cell-loaded products.

After crosslinking, all constructs were covered with the respective culture medium and incubated at 37 °C in a humidified atmosphere containing 8% CO_2_ for two weeks. The medium was exchanged three times per week.

Table 1 summarizes the detailed printing and crosslinking parameters. The bioprinting parameters were established according to the cellular needs, as listed below. The ratio between material and cells, as well as the nozzle diameter, were kept constant, and the printing pressure was adjusted as required

### 2.4. Microscopy, Image Quantification and Editing

Microscopy of the constructs was performed on days 1, 2, 4, 7 and 14 after printing using an Olympus IX83 fluorescence microscope (Olympus, Tokyo, Japan). Images were taken, merged, and quantified using the Olympus CellSens Dimension software (version 2.2, Olympus, Tokyo, Japan, 2009).

For quantification of cell survival on day 1, we counted the cells in five equal ROIs (regions of interest) in three pictures per sample and calculated the mean cell number per mm^2^.

Cell proliferation from day 4 to 14 was quantified via the mean gray value intensity of at least three raw images per condition. Respective background fluorescence was deducted by subtraction of the mean background gray value of three ROIs per image, and different exposure times were normalized based on the exposure time of 400 ms with 2fold amplification. The mean gray value intensity of the cells per image was calculated using formula 1:

mean gray value intensity = (mean gray value_total_ − mean gray value_back_) × [(exposure time_target_ × amplification_target_)/(exposure time_actual_ × amplification_actual_)]. (1)

Protrusions were analyzed with ImageJ by measuring the maximal length of three cells per time point from the center of the cell body to the utmost point of its fluorescence signal.

Graphs were designed using GraphPad Prism 8 (Graph Pad Software, San Diego, CA, USA). Brightness, contrast and intensity of the depicted images were edited with CorelDraw 2017 (Corel Corporation, Ottawa, Canada) for better perceptibility.

### 2.5. Statistics

In the bar graphs, results are expressed as mean ± s.d. Statistical analysis was performed using the GraphPad Prism 8 Software (GraphPad Software). A comparison between groups was drawn using One-way-ANOVA and subsequent Tukey’s Multiple Comparison Test. *p*-Values ≤ 0.05 were considered as statistically significant. Bars without respective labeling were not significant.

## 3. Results

### 3.1. Printing and Distribution of Melanoma Cells in Five Different Bioinks

In this study, five different bioinks were used to analyze cellular reaction, morphology and phenotype of tumor cells. The *Cellink*s are composed of alginate and nanofibrillar cellulose, one of them further coupled with RGD-peptides (*Cellink RGD*). The *GelXA* bioinks are made up of gelatin methacrylate, xanthan gum, and alginate, and one further coupled with laminin (*GelXA Laminink+*). Those modifications were chosen, as the expression of suitable receptor proteins like integrins is well described in malignant melanoma and found to be of importance for tumor growth and progression [31]. Matrigel, resembling the ECM, is composed of type IV collagen, laminin, entactin, heparan sulfate proteoglycans and growth factors. Here, we aimed to compare the naturally-derived complex matrix to artificially-produced bioinks and to define the effect of coupled ECM-peptides to the bioinks on the cellular behavior.

We printed two different fluorescent cell lines derived from melanoma metastases (Mel Im GFP, GFP; MV3dc, nuclear H2B eGFP and DsRed2; the printing parameters are given in Table 1).

**Figure 1:**
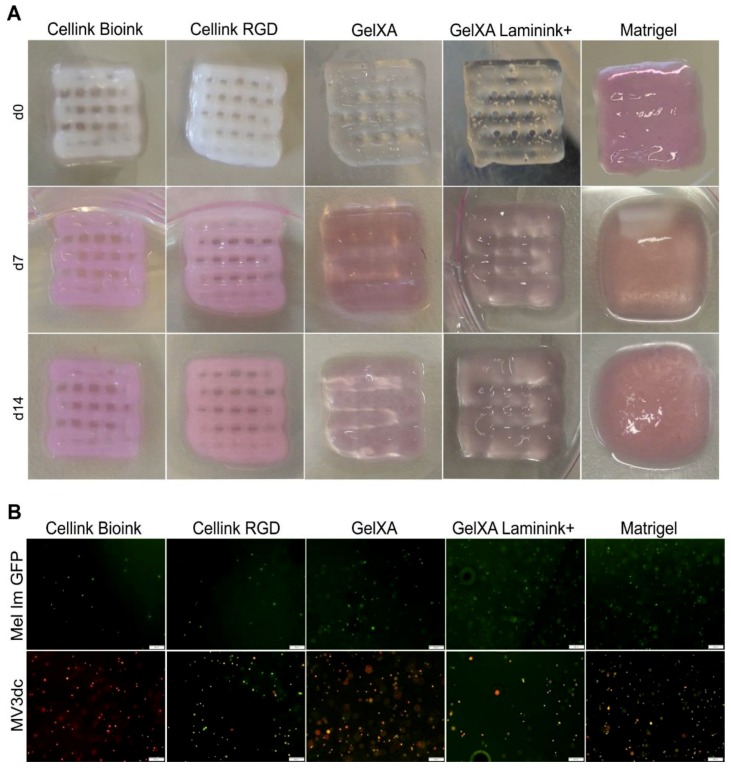
Printability of bioink and cell distribution. Constructs with an area of 1 cm^2^, three layers high, containing 10^5^ cells/mL were printed with *Cellink Bioink, Cellink RGD, GelXA, GelXA Laminink+* or Matrigel, respectively, using the Cellink+ bioprinter. (**A**) Representative macroscopic images of cell-loaded 3D printed constructs at time points d0, d7, and d14. (**B**) Representative fluorescence microscope images of melanoma cell lines Mel Im GFP (green) and MV3dc (red/green) in the respective inks 1 day after 3D printing. Scale bars represent 200 µm.

Even though we had to use different printing pressures depending on the respective bioink, all five materials were printable (Figure 1A). During cultivation, both *Cellink*s did not show any macroscopically visible changes. *GelXA*-inks displayed some minor changes based on swelling, whereas Matrigel was not able to maintain the grid structure and showed a high increase in volume primarily based on cell proliferation during the cultivation period of 14 days (Figure 1A). In all constructs, the cells were homogeneously distributed one day after printing (Figure 1B). During the cultivation time of 14 days, we did not observe sinking of the cells but nicely distributed cells at multiple levels of the constructs (data not shown).

### 3.2. Survival of Melanoma Cells in Different Bioinks

**Figure 2:**
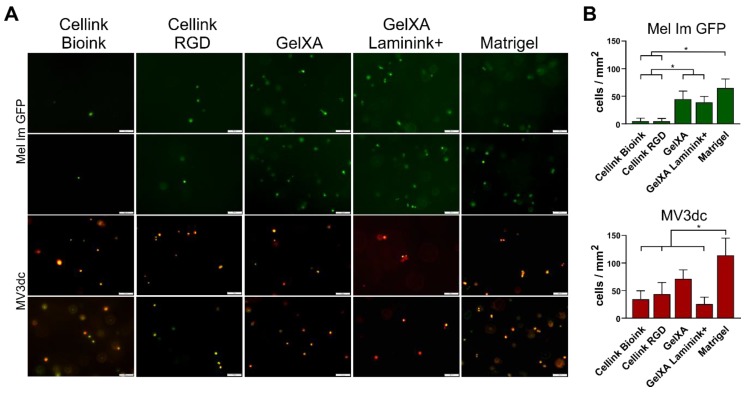
Survival of melanoma cells in the bioinks. (**A**) Two representative fluorescence microscope images of each of the cell lines Mel Im GFP and MV3dc one day after 3D printing. Both melanoma cell lines survived the bioprinting and crosslinking process in all bioinks. Scale bars represent 100 µm. (**B**) Quantification of living cells per mm^2^ in the bioinks on the day Mel Im GFP showed low amounts of living cells in both *Cellink*-based inks, a higher rate in *GelXA*-based inks, and revealed the significantly highest amount of viable cells in Matrigel. MV3dc revealed appropriate amounts of living cells in all materials, with the lowest amount in the *Cellinks* and *GelXA Laminink+*, and the significantly highest amount in Matrigel. * *p* ≤ 0.05 (One-way ANOVA).

Shear forces caused by the viscosity of the respective bioink are known to be a critical factor for cells during 3D printing. However, microscopy images revealed fluorescence signals, representing living cells after the 3D printing process (Figure 2A). The cell number for day one was analyzed (Figure 2B), as described above. In the alginate-based *Cellink*s, Mel Im GFP showed a significantly (*p* ≤ 0.05) reduced amount of living cells compared to the *GelXA*-inks where they were able to cope well with the printing process. Many viable MV3dc cells could be detected in *Cellink*s with a tendency to a higher rate of living cells in *GelXA*. The amount of living MV3dc cells was decreased by about two-thirds in *GelXA Laminink+* compared to the non-modified ink. In both cell lines, the highest cell number was detected in Matrigel (*p* ≤ 0.05).

### 3.3. Cell Morphology in Different Bioinks

**Figure 3:**
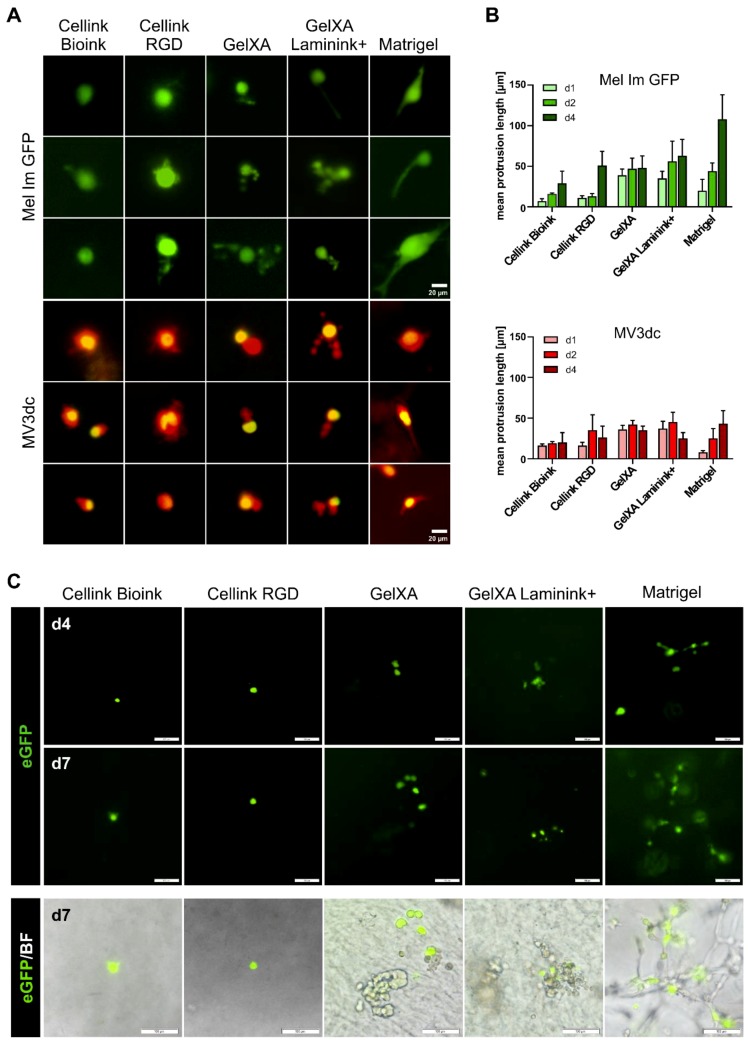
Morphology of melanoma cells in the different bioinks. (**A**) Fluorescence microscope images revealing the morphology of each three representative Mel Im GFP or MV3dc single cells on day 4, cultured in the different 3D matrices. The scale bars represent 20 µm. (**B**) Quantification of protrusion lengths (in 2D) of single cells at time points d1, d2, and d4 in all bioinks. Mel Im GFP spread and revealed increasing protrusion lengths over the observation period. Most distinct protrusions were observed in Matrigel. MV3dc cells revealed a tendency to form shorter protrusions in all bioinks, without clear time dependency of their lengths. Protrusion lengths on day 4 are not significantly different in the different materials for both cell lines (One-way ANOVA). (**C**) Fluorescence images of MCAT-eGFP transfected MV3 cells in printed constructs on day 4 and 7, the respective fluorescence/brightfield overlay images are shown for day 7. In all materials, some cells activate YAP/TAZ-TEAD, indicating cell–material interactions. The scale bars represent 100 µm.

As the five used matrices offer different adhesion cues for the cells, we expected that the melanoma cells would develop different shapes in the materials. Interestingly, the vast majority of single cells remained roundly shaped in the materials with only a small number of spreading cells in defined bioinks (Figure 3A). Protrusion lengths were analyzed for days 1, 2, and 4 after printing, as from then cells began to proliferate, and single-cell spreading could no longer be determined (Figure 3B).

For Mel Im GFP printed with the five different materials, we observed a non-significant trend of increasing protrusion lengths during the first four days of cultivation. When cultured on 2D culture plates, Mel Im GFP are spindle-shaped and develop protrusions, which are 30–90 µm in size, MV3dc are rather round-shaped, with short podia ranging between 8 and 30 µm (data not shown).

Mel Im GFP showed the least reaction in *Cellink Bioink,* where the cells remained rather small and developed only short branches of ~30 µm. In the RGD-modified *Cellink,* more branched offshoots were observed; however, maximal protrusion length after four days was only non-significantly increased to ~50 µm. In both *GelXA* and *GelXA Laminink+*, Mel Im GFP developed either short and kind of blistered or thick and elongated protrusions during the first four days of cultivation, which did not alter significantly in length (~50 µm or ~60 µm, respectively). As expected, Mel Im GFP single cells showed the highest spreading activity in Matrigel, with ~110 µm long, thick branches reaching through the material. Although we saw differences between the protrusion shapes in Matrigel compared to the other materials, the measured protrusion lengths did not vary significantly.

MV3dc single cells showed no obvious difference, whether printed in *Cellink Bioink* or *Cellink RGD*. With MV3dc cells printed in these two materials, ~20–25 µm short and blistered offshoots were observed. In *GelXA* and *GelXA Laminink+*, MV3dc single cells showed only a few small protrusions, which were already quite long on day one, but did not grow further during the following days (35 µm or 25 µm, respectively). Also, in Matrigel, only a few scattered MV3dc cells showed protrusions around 45 µm, whereas the great majority of cells rather spread towards the material by forming chain-like multi-cellular networks.

As in general the MV3dc cells were able to cope better with the printing process than the Mel Im GFP (Figure 2B), we used the MV3 cells to characterize signals related to the perception of mechanical cues and cell–matrix interactions on a molecular level. We transiently transfected MV3 cells (without stable expression of fluorescent proteins) with a reporter construct for YAP/TAZ-TEAD complex activity. YAP/TAZ activity was used as a surrogate marker for integrin-dependent binding of the cells to the ECM. It has been described previously, that upon sensation of stiff matrices, focal adhesion (FA) assembly gets promoted, leading to increased contractility of stress fibers and subsequent transport of YAP/TAZ from the cytoplasm into the nucleus, where it binds and activates the transcription factor TEAD, which in return binds MCAT-sequences on the DNA, and thereby regulates gene expression [32]. With the YAP/TAZ-TEAD complex binding to the MCAT sequence encoded on the reporter plasmid, the transfected cells express eGFP indicating FA-integrin mediated cell–matrix interactions. As before, we prepared bioinks by mixing the five materials with the transfected cells and printed the constructs as described.

The observed YAP/TAZ activity was quite low in the transfected MV3 cells in all five tested bioinks (Figure 3C). During seven days of cultivation, only very few single cells showed fluorescence signals in the *Cellinks*. In *GelXA* bioinks and Matrigel, which all contained integrin-binding motifs, we detected more cells delivering an eGFP signal. However, neither the cells within the RGD-coupled matrix nor cells in *GelXA* coupled with laminins showed an increased amount of fluorescent cells compared to cells in the respective non-modified biomaterials.

### 3.4. Proliferation of Melanoma Cells in Different Bioinks

**Figure 4:**
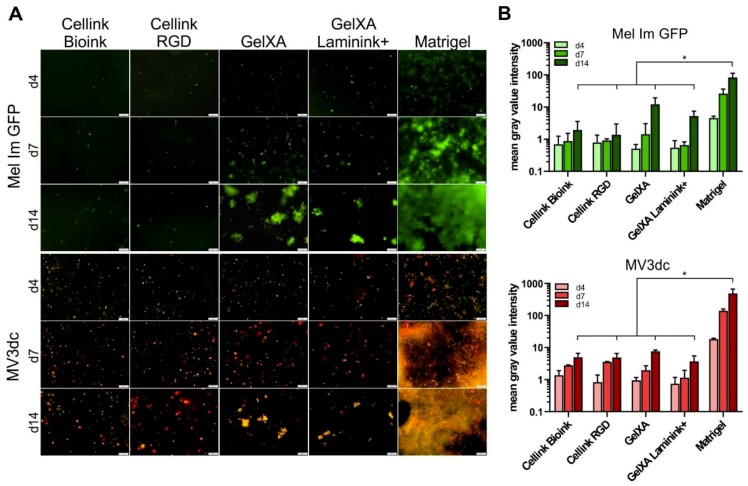
Tumor cell proliferation in bioinks over 14 days of culture. (**A**) Representative fluorescence images showing the proliferation of Mel Im GFP and MV3dc on d4, d7, and d14 after being printed with *Cellink Bioink*, *Cellink RGD*, *GelXA*, *GelXA Laminink+* and Matrigel. Mel Im GFP and MV3dc showed hardly any growth or proliferation *in Cellink Bioink* and *Cellink RGD* but formed irregular clusters in *GelXA* and *GelXA Laminink+*. In Matrigel, cells proliferated quickly, Mel Im GFP grew in clusters, which fused together with time, whereas MV3dc cells formed dense chain-like networks throughout the material. Scale bars represent 200 µm. (**B**) Quantification of proliferation by determination of the mean gray value intensities of at least three fluorescence images per time point plotted logarithmically to emphasize the strongest proliferation of both cell types in Matrigel. The statistical comparison presents the significant difference of mean gray value intensities in Matrigel on day 14 with the other materials on that day. The same significance was observed in comparing day 4 or day 7; however, due to clarity reasons, these significances are not shown in the graph. * *p* ≤ 0.05 (One-way ANOVA).

As Mel Im GFP and MV3dc were shown to survive the bioprinting process, we analyzed the cell proliferation in the different bioinks over time. The cells displayed clearly distinguishable proliferation behavior comparing Matrigel, the alginate-based (*Cellink*) and the gelatin methacrylate-based (*GelXA*) bioink over time (Figure 4A,B). (Significances of cell proliferation from day 4 to 14 within the different materials are displayed in Table 2.)

Mel Im GFP cells did not proliferate in the *Cellink Bioink* and *Cellink RGD* but were able to survive within the material over the culturing time of 14 days. No cell doubling could be observed in these materials, indicating a lack of proliferation. MV3dc cells showed a tendency to be able to cope better with the printing process with *Cellink*s (see Figure 2A,B), and also revealed an approximately five-fold increase in mean gray value intensities from day 4 to 14 of cultivation, indicating not only long-term survival but also moderate proliferation in the material (*p* ≤ 0.05, Table 2).

In *GelXA* constructs, we observed a stronger increase in mean gray value intensity for both Mel Im GFP and MV3dc during the culture time compared to *Cellink*s. Already after seven days in culture, cells had clearly proliferated and formed small clusters, which grew on to form irregularly sized and shaped clusters after 14 days (*p* ≤ 0.05, Table 2).

Interestingly, neither the modification of *Cellink Bioink* with RGD-sequences for integrin binding nor providing *GelXA* coupled with a mixture of different laminins revealed a beneficial effect for cell proliferation after 14 days. Whereas both cell types showed comparable proliferation in *Cellink Bioink* and *Cellink RGD*, they even showed a trend towards slightly decreased proliferation in the laminin-modified *GelXA*.

In Matrigel, cells proliferated quickly, forming clusters or loose accumulations of cells during the first week of cultivation and growing out to dense networks pervading the material after two weeks. This led to very high mean gray value intensities in the quantification, depicting the significantly highest proliferation rates after 4, 7 and 14 days in comparison to the other materials at the respective time points (Figure 4). Additionally, we observed no significant differences between the mean gray value intensities of Mel Im GFP or MV3dc in Cellink Bioink, Cellink RGD, GelXA and GelXA Laminink+ at any of the analyzed time points, namely after 4, 7 or 14 days in culture.

The proliferation behavior we observed microscopically also becomes visible by looking at the constructs themselves (Figure 1A). The *Cellink*s, in which cells did not grow or divide, maintained their shape over time. *GelXA* inks seemed to swell a little bit during the culturing phase. The Matrigel constructs, in which cells showed the highest proliferation rates, were clearly swollen after seven days and were found to be burst open after 14 days in culture.

## 4. Discussion

Melanoma is a highly malignant tumor type, responsible for 90% of all deaths related to skin tumors. It is often used as a model tumor for the establishment of novel therapies; however, many aspects of tumor progression are still unknown. Due to the high complexity of the natural systems, common 2D culture methods might not be sufficient to elucidate the various processes that happen during cancer development and progression. The tumor microenvironment (TME) is composed of different cell types, including stem cells, fibroblasts and immune cells, blood vessels, soluble factors and the ECM, which all contribute to cellular behavior and thereby to cancer disease. Fibroblasts, for example, are known to release cytokines influencing the growth and differentiation of other cell types within the tumor mass and trigger angiogenesis, leading to improved nutrient supply and survival of the tumor mass. Moreover, they promote ECM remodeling in tumors, leading to the characteristic stiff tumor matrix, which, in turn, plays a crucial role in the regulation of cell proliferation [33], migration [34], differentiation [35] and survival [36]. These are only a few factors contributing to cellular behavior in their natural niche, pointing out the importance for more physiological 3D culture systems, which enable the consideration of the physical TME [3,37]. Three-dimensional cell culture systems enable the addition of stromal cell types, selected matrix proteins, growth factors and so on, allowing for the control of the microenvironment sensed by the embedded cells.

Three-dimensional bioprinting goes even one step further by enabling discrete cell distribution and precise arrangement and is, therefore, of great interest when it comes to the development of highly defined 3D models, which might help to decipher the complex physical interactions happening during cancer disease and treatment.

We were able to show the printability of the two melanoma cell lines Mel Im GFP and MV3dc using the biomaterials *Cellink Bioink*, *Cellink RGD*, *GelXA* and *GelXA Laminink+*, which are in combination with cells by definition, as bioinks are supposed to offer reliable cell compatibility as well as printability and stability over longer periods of time. Matrigel, the current gold standard for a rich physiological ECM, but not classical bioink as it lacks shape fidelity and long-term stability, has been used in the field of biofabrication for some time. However, in published studies, Matrigel was used basically as a coating material for 3D printed scaffolds, as the physiological component of a printable matrix mixture with hydrogels like alginate, gelatin or agarose or it was printed into predefined molds (e.g., [38,39,40,41,42]). Here, we developed a method to print pure Matrigel, which, although revealing the poorest printability in comparison with the other inks, was printable with the tumor cells and was able to keep a 3D structure over the culturing period of 14 days without dissolving in the medium. Equal distribution of the cells was observed in all 3D multilayer-constructs, which is often problematic in usual 3D cell culture and is a major advantage of 3D bioprinting.

It is known that cells are exposed to critical pressure and shear stress during 3D printing [43] and also sense unfavorable circumstances during crosslinking procedures using a CaCl_2_ solution or UV-light. However, we observed that cells were able to survive the production process in all tested materials, but to a different extent. The more viscous the bioink, the stronger the shear forces acting on the cells [44]. Therefore, it is not surprising that cells showed the highest amounts of living cells in Matrigel, followed by the *GelXA* inks and *Cellink*s. When cells were mixed with Matrigel, it was relatively liquid, and even after pre-gelling, printing pressure could be kept quite low between 3 and 7 kPa. *Cellink Bioink* and *RGD* were way more viscous from the beginning, so the cells sensed higher shear forces during mixing and printing with approximately 20 kPa. *GelXA*-inks were liquid during the cell mixing step as well, but got a lot stiffer during pre-gelling, and had to be printed with approximately 25 kPa. Commercial bioinks were all crosslinked with CaCl_2_, which might also lead to decreased amounts of living cells in comparison to Matrigel. Ca^2+^ is a highly important ion participating in the regulation of cellular functions [45,46,47,48], which might get disrupted by the treatment with CaCl_2_, as shown by Cao et al. [49]. On the contrary, due to its high protein content, no chemical crosslinking was needed for Matrigel, as it hardens upon temperature treatment with cell-compatible 37 °C.

Despite these observations, not only the properties of the bioink but also the used cell line seemed to have a significant influence on cell survival. Even though we used two melanoma metastasis-derived cell lines, we found that the Mel Im GFP cells were more sensitive to printing with *Cellink*s compared to MV3dc, which survived the printing process notably better. These results indicate that material properties and the printing process have to be adjusted for each single cell line, which is supposed to be used. This is consistent with our observations concerning the cellular activities in the printed constructs during 14 days of culture, which revealed that not only the material composition decides over cell fate but also the used cell line itself.

An important method to manipulate the cellular behavior in 3D cultures is the modification of the matrix material with defined molecules, aiding attachment, proliferation and overall viability of the cells. These molecules (e.g., laminin) are tissue-specific in vivo and should be adjusted for the desired applications. As reviewed, for example, by Kuphal et al., the expression pattern of the cell surface receptors called integrins is well characterized for malignant melanoma [31]. Several studies revealed the importance of certain integrins for adhesion, migration and matrix reorganization in malignant melanoma [50,51,52]. Here, we chose the RGD-peptide and a laminin blend as modification molecules, as the expression of suitable receptor proteins in malignant melanoma has been described before [31,53,54].

In the alginate-based inks, the cells were able to survive, indicating that they were supplied with nutrients from the medium. However, the material might be too dense for proper cell proliferation. *Cellink*s contain nanofibrillar cellulose, which is cell compatible and beneficial for reliable printability and long-term stability of the printed constructs [55]. Cellulose cannot be digested by cellular enzymes like matrix-metalloproteases (MMPs), just as the main component alginate, and might, therefore, trap the used cells and prevent them from proliferating. Contrary to our expectation of matrix-dependent cell morphology, we hardly saw differences like the distinguishable formation of protrusions comparing the RGD-modified and the non-modified *Cellink*. The spatial hindrances, which the cells sense in a non-digestible matrix, might also prevent proper spreading in the *Cellink RGD*, as the offered RGD sequences might not be reachable for the cells and only thin, branched protrusions can be formed. It has to be considered that, here, we quantified the protrusion lengths only in two axes by microscopy and might have thereby missed protrusions reaching towards upper or lower levels. Nevertheless, we claim that our images would reveal increased or varied protrusions, as performed by other groups before [56,57]. Jia et al. printed human adipose-derived stem cells with oxidized and non-oxidized alginate coupled with RGD and were also not able to detect proper spreading in the non-oxidized construct, whereas in the oxidized version, cells were able to develop protrusions. They suggest that this might be due to the low porosity and degradability of the alginate, which can be improved by oxidation of the material [56,58]. Another option is the addition of gelatin to the alginate, which does not only offer RGD binding sites by nature but is also degradable and will increase pore size of the construct over culture time [59].

This gelatin content might also be the reason why, in the gelatin methacrylate-based bioinks, cells were able to recognize the material and spread. Cells proliferated in clusters, which were irregular in size and shape. As mentioned before, gelatin is recognized by MMPs and can be digested, resulting in more space for proliferating cells [60]. Surprisingly, despite its known importance for cell differentiation, cell shape, movement and survival [61], the modification of the basic material with a laminin-mixture did not have any significant effect on cellular behavior, neither concerning proliferation, nor cell spreading or survival. It has to be considered, that there are 16 isoforms of laminin, all of which are bound by their receptors with tissue-dependent specificity [62]. Interestingly, the major laminin-binding integrin heterodimer *α7β1* is known to be present in primary and metastatic melanoma tissues, but cannot be detected in in vitro cultures [31]. However, there are further integrins like *αvβ3* or *α3β1,* which are highly expressed in melanoma metastases and should be able to recognize certain laminin structures. Maybe the incorporated laminin blend contains too many non-relevant laminin isoforms for the melanoma cells that cannot be targeted by the present cell surface receptors.

We detected the highest activity concerning proliferation and spreading in the gold standard Matrigel, which offers not only several cues for the cells to attach but also to digest the matrix while growing and proliferating throughout the matrix. Interestingly, Mel Im GFP single cells developed multiple sweeping branches, which were overgrown by the proliferating round shaped cells after two weeks of culture, while MV3dc cells developed only short protrusions when lying separately but were able to elongate, spread and form chains throughout the material when they got in contact with neighboring cells. This observation underlines the cell type specificity and the varying reactions of different cell types to a certain range of cues as delivered here by the Matrigel.

As YAP/TAZ are known mechanosensing and -transducing players in regulating tumor growth [63], we wanted to characterize their participation in mechanosensation in our printed constructs. We received signals from the cells in all five materials; however, compared to the *Cellink*s, we observed increased YAP/TAZ-TEAD activity signals in *GelXA*-inks and Matrigel. As already seen for the cell spreading and as described above, this is probably due to increased integrin-matrix binding, as these materials offer more targets for the integrin receptors. As mentioned before, Matrigel provides the cells with RGD-peptides, laminins, collagen, fibronectin, and many more molecules, where a huge set of different types of integrins, which are highly expressed in melanoma cells, can attach to [31]. On the contrary, *Cellink RGD* offers only the RGD-peptide, which is recognized by a smaller set of integrins only.

All three tested biomaterial bases (*Cellink Bioink*, *GelXA*, Matrigel) exhibit characteristics, which are useful for the investigation of different aspects of tumor development and progression. *Cellink Bioink*, in which cells were hardly showing proliferation or morphological changes but were able to survive, might be useful for investigating, for example, the barely understood mechanism of tumor dormancy. Here, triggers of cell proliferation, for example, by the addition of certain growth factors or by using different material dilutions, could be determined to understand how an escape from dormancy is mediated. However, clear bioinks would be preferred to the opaque *Cellink* series to facilitate cell observation. *GelXA* and, especially, the printed Matrigel, in which cells were able to proliferate, pose suitable platforms for drug testing in a 3D microenvironment. Homogeneous constructs can be printed easily and can be treated with different substances and conditions. Further developments could include the printing of cocultures of melanoma cells with stromal cells or other cell types of the tumor microenvironment in a defined spatial organization to take another step towards a physiological melanoma tumor model. Consequently, the definition of the most suited bioink cannot be drawn in a general way, but largely depends on different aspects, including cell type, the experimental question and the desired application.

Taken together, our experiments revealed the printability of two melanoma cell lines in four commercially available bioinks and Matrigel. Both cell lines survived the printing process and the following two weeks of cultivation, revealing different proliferation and spreading behavior depending on basic material composition. Contrary to our expectations, cell survival, morphology and proliferation did not benefit from material modifications with RGD or laminins. Upon exposure to the same environmental conditions, we observed differences in cell survival, morphology and proliferation of the two melanoma cell lines, outlining the difficulties for the development of appropriate bioinks.

We conclude that 3D bioprinting requires a precise understanding of cell and material properties, cell–material interactions as well as cell and material behavior during the printing process, which we currently lack, in order to figure out how to control certain cells with certain cues, before we can start to develop valid 3D models or even tissues. It will be of great interest to further investigate the molecular signaling, which is happening in the cells embedded in the constructs, in order to define molecules involved in signal transduction to get closer to being able to control and manipulate cellular reactions in defined 3D structures.

## Figures and Tables

**Table 1:** Printing parameters.

Material		Mixing Parameters		Printing Parameters			Crosslinking Parameters	
Trade name	Composition	Ratio *	Material temp. [°C]	Nozzle Ø [gauge]	Pressure [kPa]	Bioink temp. [°C]	Method	Time [min]
*Cellink Bioink*	alginate, nanofibrillar cellulose	11:1	22–24	22	11–22	22–24	50 mM CaCl_2_	5
*Cellink RGD*	alginate, nanofibrillar cellulose, RGD-modification	11:1	22–24	22	20–24	22–24	50 mM CaCl_2_	5
*GelXA*	gelatin methacrylate, xanthan gum, alginate	11:1	35	22	20–47	22–24	50 mM CaCl_2_	5
*GelXA Laminink+*	gelatin methacrylate, xanthan gum, alginate, laminin-modification	11:1	35	22	20–32	22–24	50 mM CaCl_2_	5
Matrigel	laminin, collagen IV, heparan sulfate proteoglycans, entactin/nidogen, growth factors	11:1	4	22	3–7	22–24	37 °C	30

* ratio of biomaterial to cell suspension in the respective cell culture medium.

**Table 2:** List of statistical significances of cell proliferation comparing day 4 to day 14 after printing in the respective bioinks.

	Cellink Bioink	Cellink RGD	GelXA	GelXA Laminink+	Matrigel
**Mel Im GFP**	n.s.	n.s.	*	*	*
**MV3dc**	*	*	*	*	*

* *p* ≤ 0.05; n.s. = not significant (One-way ANOVA).
